# Combination application of microbial agent and lime application improves soil nutrients and soil fungi community, producing good quality tobacco

**DOI:** 10.3389/fmicb.2025.1615412

**Published:** 2025-07-22

**Authors:** Jingtao Zhang, Chao Li, Kai Xia, Kaikai Cheng, Shanhu Guo, Wenzheng Pan, Lingling Liu, Ke Wang, Li Wen, Haiming Tang

**Affiliations:** ^1^China Tobacco Hunan Industrial Co., Ltd., Changsha, China; ^2^Hunan Cultivated Land and Agricultural Eco-Environment Institute, Changsha, China; ^3^Yunnan Microbial Fermentation Engineering Research Center Co., Ltd., Kunming, China

**Keywords:** agent addition, soil fungi, lime, soil fertility, continuous cropping barrier

## Abstract

Agent addition is increasingly recognized as a crucial strategy for improving soil health in tobacco cultivation. However, its impacts on soil microbial community and plant growth differ depending on soil conditions. In this study, tobacco soil was collected in Wu Ding County, Yunnan Province, under four distinct agent addition treatments, simply chemical fertilizer (T1), microbial agent + chemical fertilizer (T2), lime + chemical fertilizer (T3), and microbial agent + lime + chemical fertilizer (T4). Using the Illumina high-throughput sequencing platform and fungal ribosomal DNA internal transcribed spacer 1 to analyze the distribution characteristics of fungal communities in tobacco soil. Soil nutrient indicators (pH, SOC, TN and AP) were considerably higher in T4 than in T1. In comparison to T1, agent addition boosted the agronomic characteristics such as maximum leaf length, maximum leaf width, stem girth, and leaf number. T4 treatment facilitated the harmonization of chemical composition of tobacco leaves and greatly increased tobacco yield by 8.94% than T1. The application of T4 resulted in a reduction of fungal diversity. *Ascomycota* was the most dominant phylum across all soil and agent applications distinctly shifting the soil fungal community diversity. Furthermore, certain beneficial fungi were obviously accumulated, but the potentially pathogenic fungi were noticeably reduced or absent in T4. An explicit enrichment of saprotrophic fungi in T4 is predicated by the FUNGuild function. Soil nutrients were extremely significant and relevant in relation to the fungal community structure. In summary, we propose that T4 treatment could be an effective strategy to alleviate the continuous cropping barrier in tobacco cultivation since it eliminates soil acidity, improves soil nutrients, and modifies the soil microbial community structure, thereby improving the plant growth and increasing the yield of tobacco.

## 1 Introduction

Tobacco is one of the most important industrial crops that is intolerant to continuous cultivation (Chen et al., 2018; Bai et al., 2019). Extensive studies have identified several key factors contributing to cultivation challenges: nutrients depletion in soil, degradation of soil physical and chemical characteristics, alterations in soil microbial community structure, and plant autotoxicity (Kayikcioglu et al., 2020; Chen et al., 2022). Based on a survey, short-term continuous cropping can lead to substantial tobacco yield losses ranging from 20 to 100%, coupled with diminished cigarette quality primarily due to soil-borne pathogens (Chen et al., 2016). Consequently, developing sustainable agricultural strategies to mitigate these continuous cropping barriers has become imperative for maintaining crop productivity and quality.

Several strategies have been implemented to deal with the challenges associated with continuous cultivation. These include the utilization of inorganic, organic, and biological fertilizers (Wu et al., 2014). Fertilizer, mostly chemical, is frequently utilized to overcome obstacles to continuous cultivation. However, intensive chemical fertilization has been attributed to environment challenges like water eutrophication and elevated emission of greenhouse gases, as well as aggravating major soil degradation problems consisting soil acidification, soil nutrient depletion and microbial biomass and diversity decline (Snyder et al., 2009; Wang et al., 2020; Li C. et al., 2022; Zhang et al., 2024). These circumstances highlight the urgent need for sustainable soil fertility management strategies that enhance productivity while minimizing ecological costs. Different from sole chemical fertilization, agent addition, e.g., microbial agent addition and lime application, have been developed and emerged in agricultural cultivation (Xin et al., 2022; Liu et al., 2023; Ahsan et al., 2024). Microbial agents contain active and functional microorganisms that improve crop production and restore soil equilibrium by reducing compaction, chemical fertilizer effects, and soil- borne diseases (Li Q. et al., 2022). Lime application is a common and effective strategy to ameliorate soil acidification for increasing soil fertility and plant productivity (Deus et al., 2020; Li S. et al., 2022). Moreover, lime added to soils can release a large amount of heat energy when hydrolyzed, effectively eliminating pathogenic microbe and root-knot nematodes (Li S. et al., 2022). These application may provide favorable conditions for soil microorganism activity and biological processes (Gatch and du Toit, 2017; Li et al., 2021; Li S. et al., 2022). For instance, Li et al. (2021) discovered that the quantity of lime addition increased the structure, function and diversity index of soil microbial community. While Yin et al. (2021) and Narendrula-Kotha and Nkongolo (2017) revealed that lime addition had no effect on the diversity of soil fungus communities. Therefore, the inconsistency results indicated that more research is needed to evaluate the fluctuation of soil microbial communities in acid soils when microbial agents and lime are applied.

Soil microbes play a crucial role in plant growth and health and are involved in many key processes of soil ecosystems including soil nutrient cycling (Brussaard et al., 2007; Berg, 2009; Sahu et al., 2019; Yang et al., 2020). The composition, diversity and function capacities of soil microbes are essential in sustaining soil health and ecosystems resilience (Sahu et al., 2019; Yang et al., 2020). It is well acknowledged that the decline in soil microbial community diversity and enrichment of the dominant pathogenic species has resulted from continuous cropping (Shi et al., 2020; Wen et al., 2024). A focus on the interaction between soil microbial communities and individual fertilization strategy may lead to bias in determining the effectiveness of fertilization strategies in tobacco cultivation. Hence, understanding the knowledge of soil microbial communities under different agent addition strategies may assist with determining fertilization strategies that are likely to minimize continuous cropping obstacles.

The Yunnan tobacco producing area, which has been cultivating tobacco for over 170 years, represents China’s strong-flavored flue-cured tobacco. In recent years, the shortage of land resources has led to a severe succession of crops, causing soil degradation and poor nutrient availability for tobacco plants. Thus, over fertilization practices, particularly chemical fertilizer, are widely implemented to pursue high yields of tobacco. However, long-term chemical fertilizer application has been led to severe soil acidification, soil nutrient reduction, crop yield and quality decline and frequent soil-borne diseases, which have become a major problem restricting the sustainable development of tobacco industry (Zhang et al., 2022). As a results, there is an urgent need to improve fertilization strategies to improve the yield of flue-cured tobacco and maintain sustainable development of tobacco industry. Currently, combination application of fertilizer and agent addition have implemented in tobacco planting, which aims to optimize soil condition of tobacco growth and in turn led to increased yield and improve quality. The modification of soil microbial community has been verified as one of the important factors that influence crop growth. Thus, the objective of this study were (1) to evaluate the effects of different agent addition on soil properties and growth of tobacco and (2) compare the changes of soil microbial community among different agent additions. Understanding the impact of agent addition on the soil condition of tobacco planting will provide theoretical guidance for rational adoption of farming mode and sustainable utilization of tobacco industry.

## 2 Materials and methods sampling and methods

### 2.1 Site description

The study site was located in Wuding County (25°20′–26°11′N, 101°55′–102° 29′E) of Yunnan Province. This region is a typical subtropical plateau monsoon climate with a mean annual temperature of 15.1°C and an annual precipitation of 1,000 mm. Based on the Chinese Soil Taxonomy, the soil at the site is classified as Brown Soil. This area were carried out for tobacco cultivation for about 40 years and with conventional chemical fertilization practice. The field experiment employed a randomized block design with three replicates across four treatments, including four treatments: only conventional chemical fertilizer (T1), combination application of microbial agent and chemical fertilizer (T2), combination application of lime and chemical fertilizer (T3), and combination application of microbial agent and lime with chemical fertilizer (T4). Each treatment set has triple plots (20 m × 15 m). The tobacco variety was Yunyan 87, with 110 cm row spacing and 50 cm plant spacing. Seedlings were transplanted on May 10, and the harvest was completed on October 27, 2023. All additional agricultural practices adhered to the local management standards for cultivating high-quality tobacco leaves.

In this study, tobacco (*Nicotiana tabacum* L.) was planted in April 2023 under the following land management practices: four fertilization regimes were applied during the growth period, and the soil was ploughed 25 cm deep. A base fertilizer (compound fertilizer: N-P_2_O_5_-K_2_O = 15-15-18, 300 kg hm^−2^) was used initially, transplanting 10–15 days and 20–25 days, respectively, 225 kg hm^−2^ compound fertilizer and 75 kg hm^−2^ potassium nitrate were added during the seeding stage. After 35 days of transplanting, 150 kg hm^−2^ potassium sulfate fertilizer and 225 kg hm^−2^ compound fertilizer were added with tillage. *Bacillus subtilis* strains that were extremely active were among the ingredients of the microbial agent. The total effective number of viable bacteria is >5.0 × 10^8^ CFU mL^−1^. Microbial agents (30 kg hm^−2^) were added at transplanting and the pendulum stage at a diluted 400 times with water (15 L hm^−2^), respectively. Lime addition was applied as powder into soil with 1,500 kg hm^−2^ before tobacco transplanting.

### 2.2 Sampling collection and measurement

#### 2.2.1 Soil sampling and methods of soil physiochemical properties

Soil sampling (0–20 cm) was conducted in August 2023, at the crops mature stage for tobacco. A random collection of 8 points in each plot was mixed as one sample. After removing stones and roots, a portion of fresh soil sample was stored at −80°C for DNA extraction. The remaining portion of the soil sample was air-dried and passed through a 0.15 mm mesh to measure soil physicochemical properties, including soil pH, soil organic carbon (SOC), total nitrogen (TN), total phosphorus (TP), and total potassium (TK), available nitrogen (AN), available phosphorus (AP), and available potassium (AK). All methods for detecting soil-based properties have been performed previously (Wen et al., 2023; Wen et al., 2024).

#### 2.2.2 Tobacco sample collection and determination

Before harvesting the tobacco in August 2023, five representative tobacco plants were randomly selected from each plot to observe height, stem girth, leaf number, pitch, leaf length and leaf width according to the standard method of YC/T 142-2010. The selected plants were then collected all leaves and weighted to measure yield. In addition, the leaves of tobacco were collected and washed with tap water and deionized water, and then placed into the oven at 105°C for 15 min and then at 65°C until constant weight. The chemical properties of tobacco leaves, including total sugar, reducing sugar, total nitrogen, chloride and potassium, were determined by continuous flow analyzer (AutoAnalyzer 3, Brown Ruby, Germany) based on the standard method of YC/T 159-2019 and YC/T 161-2002.

#### 2.2.3 DNA extraction, PCR amplification and amplicon high throughput sequencing

The DNA extraction and ITS rRNA amplification sequencing were performed by Genesky Biotechnologies Inc., Shanghai, 201,315 (China), and the method was descried by Wen et al. (2024). We extracted soil DNA from 0.25 g of soil (dry weight) using a FastDNA^®^ SPIN Kit for Soil (MP Biomedicals, Santa Ana, CA). Each composite soil sample was extracted in triplicate. The integrity of genomic DNA was detected through agarose gel electrophoresis, and the concentration and purity of genomic DNA were detected through the NanoDrop 2000 and Qubit 3.0 spectrophotometer (Thermo Fisher Scientific, United States). DNA from the soil samples was used as a template for PCR amplification. As positive control, standard genomic DNA of fungi was amplified in triplicate. The PCR products were purified with Agencourt AMPure XPPCR Purification Beads (Beckman Coulter, United States) to assess their specificity. The primers used for the amplification of the ITS1 region were ITS1F (5′-CTTGGTCATTTAGAGGAAGTAA-3′)/ITS2R(5′-GCTGCGTTCTTCATCGATGC-3′) and then sequenced using Illumina NovaSeq 6000 sequencing (Illumina, United States) (Gardes and Bruns, 1993; Usyk et al., 2017; Wen et al., 2024). The PCR mixture consisted of 1 μL of 10 × Toptaq buffer, 0.8uL of 2.5 mM dNTPs, 0.3 μL of F/R primer (10 μM), 0.2 μL of Toptaq DNA polymerase (Transgen, China), 3 μL template DNA, and up to 10 μL ddH_2_O. The PCR cycle conditions were initial denaturation at 94°C for 3 min, 94°C for 30 s, 55°C for 30 s, and 72°C for 1 min; 25–27 cycles of denaturing at 72°C for 10 min, and hold at 4°C. The purified products were indexed in the 16S V4–V5 library. The library quality was assessed on the Qubit@3.0 Fluorometer (Thermo Scientific, Wilmington, United States) and Agilent 2100 Bioanalyzer (Agilent Technologies, United States) systems. Finally, the purified amplifications from all the samples were pooled in equimolar concentrations, and then the pooled library was sequenced on a NovaSeq 6000 platform (Illumina, United States), SP-Xp (PE250) using a 2 × 250-paired-end sequencing kit (250 bp).

Paired-end reads were assembled using FLASH to obtain raw tags. Accession PRJNA1213986The raw read sequences were processed in the Quantitative Insights Into Microbial Ecology (QIIME) toolkit (Bolyen et al., 2019). The adaptor and primer sequences were trimmed using the cutadapt plugin. High-throughput sequencing data were clustered into operational taxonomic units (OTUs) at a 97% similarity level using the UPARSE pipeline, and the chimeric sequences were identified and removed. The DADA2 plugin was used for quality control and to identify amplicon sequence variants (ASVs) (Callahan et al., 2016). Taxonomic assignments of ASV representative sequences were performed with a confidence threshold of 0.6 by a pre-trained Naive Bayes classifier that was trained on the UNITE (version 8.2).

### 2.3 Statistical analysis

Before statistical analyses, all the dependent variables were tested for normality and natural log-transformed when necessary. One-way ANOVA with LSD test was conducted using SPSS 20.0 for Windows (SPSS Inc., Chicago, IL, United States) to determine significant differences among different treatments (*p* < 0.05). One-way analysis of variances (ANOVA) with least significant difference (LSD) test was performed to investigate the significant difference in soil and plant properties across fertilization treatments. The relative abundance was determined by the number of sequences affiliated with the same phylogenetic groups divided by the total number of the target phyla or genera per sample. Soil microbial diversity indexes, i.e., Chao 1, ACE, Shannon and Simpson index, were calculated to compare the fungi community alpha diversity for each treatment. The Venn analysis was performed to compare the fungal composition. Principal coordinates analysis (PCoA) and nonmetric multidimensional scaling (NMDS) were performed to detect the beta diversity between individual samples. Linear discriminant analysis (LDA) effect size (LEfSe) was used to elucidate the biomarkers in each treatment. Those with an LDA ≥2.0 were considered to be important biomarkers in each treatment. Redundancy analysis (RDA) with a forward selection option and Monte Carlo permutations test (9,999 permutations) and heatmap correlation analysis was conducted to select the significant explanatory variables (soil properties) contributing significantly (*p* < 0.05) to soil fungi microbial community variation. The RDA was performed using the Canoco 5.0 software.

## 3 Results

### 3.1 Soil physicochemical properties, agronomic character, chemical constituents and yield of tobacco

Among 10 soil properties, only soil pH, SOC, TN, AP, and N:P were significantly varied among four treatments (Table 1, *p* < 0.05). Compared with T1 treatment, other three treatments increased soil pH by 0.45, 0.41, and 0.31 units, respectively (Table 1). T3 and T4 treatments significantly increased the content of SOC and TN (Table 1, *p* < 0.05). In addition, T4 treatment significantly increased AP content and N:P ratio, and these two indexes were insignificantly shifted among T1, T3, and T4 treatments (Table 1).

**Table 1:** Soil properties under four agent addition treatments.

Soil properties	T1	T2	T3	T4
pH	4.92 (0.04)b	5.23 (0.03)a	5.33 (0.08)a	5.37 (0.14)a
SOC, g kg^−1^	16.83 (1.00)c	21.46 (0.42)a	21.34 (0.43)a	17.82 (2.07)ab
TN, g kg^−1^	1.91 (0.02)c	2.49 (0.11)a	2.25 (0.09)ab	2.06 (0.10)bc
TP, g kg^−1^	0.78 (0.02)	0.78 (0.05)	0.78 (0.06)	0.81 (0.02)
TK, g kg^−1^	21.34 (0.38)a	20.92 (0.60)ab	21.39 (0.02)a	19.74 (0.50)b
AN, mg kg^−1^	180.81 (10.25)	203.12 (10.51)	212.34 (13.05)	196.64 (9.97)
AP, mg kg^−1^	14.16 (2.93)b	54.19 (12.20)a	32.16 (7.69)b	15.85 (3.47)b
C:N	8.80 (0.51)	8.65 (0.49)	9.51 (0.50)	8.59 (0.57)
C:P	21.64 (0.68)	27.68 (1.41)	27.99 (3.10)	21.97 (2.35)
N:P	2.47 (0.06)b	3.21 (0.19)a	2.93 (0.19)ab	2.54 (0.11)b

Values are presented as mean± standard error. Different lowercase letters present significant difference among four agent addition treatments at *p* < 0.05. T1, only chemical fertilizer; T2, microbial agent + chemical fertilizer; T3, lime + chemical fertilizer; T4, microbial agent + lime + chemical fertilizer. The same below.

In terms of tobacco growth and development, T3 and T4 increased the plant height by 0.65 and 2.03% compared to T1, respectively (Table 2). In comparison to T1, fertilization practices increased 4.41–6.42% for maximum leaf length, 4.25–10.97% for maximum leaf width, 10.01–17.13% for stem girth, and 4.65–7.91% for leave number, respectively (Table 2). T4 treatment resulted in an 8.94% increase in tobacco leaf yield compared to T1 treatments, whereas T2 and T3 treatments had comparable results (Table 2).

**Table 2:** Tobacco agronomic characters and leaf yield under four agent addition treatments.

Agronomic characters	T1	T2	T3	T4
Height, cm	129.80 (7.10)	129.92 (8.92)	130.65 (14.58)	132.44 (13.49)
Maximum leaf length, cm	75.07 (0.04)	78.38 (0.05)	79.72 (0.09)	79.89 (0.08)
Maximum leaf width, cm	29.91 (0.02)	31.18 (0.02)	32.69 (0.02)	33.19 (0.03)
Stem girth, cm	9.69 (0.01)	10.66 (0.01)	11.35(0.02)	11.06 (0.04)
Pitch, cm	6.65 (0.01)	6.89 (0.02)	7.12 (0.02)	7.1 (0.02)
Leaves number	21.5 (0.52)	22.5 (0.04)	23.2 (1.00)	23.1 (1.15)
Yield, t hm^−2^	2.46 (0.05)b	2.48 (0.05)ab	2.57 (0.1)ab	2.68 (0.09)a

Values are presented as mean± standard error. Different lowercase letters present significant difference among four agent addition treatments at *p* < 0.05.

As shown in Table 3. Microbial agent and/or lime addition significantly increased the content of total sugar, reducing sugar and total nitrogen of tobacco upper leave (Table 3, *p* < 0.05). And these were not achieve significant variance in T2, T3, and T4 treatments. Compared with T1, T3 treatment significant decreased the potassium content of tobacco (Table 3, *p* < 0.05). Further shown that the ratio of potassium to chloride was notably lower in T2, T3, and T4 compared to that in T1 (Table 3, *p* < 0.05). Nevertheless, the higher ratio of reducing sugar to total sugar was existed in T3 and lower value in T2.

**Table 3:** Chemical composition of tobacco leaves under four treatments.

Chemical compositions	T1	T2	T3	T4
Total sugar, %	28.81b	33.14a	31.94a	33.38a
Reducing sugar, %	24.28b	26.77ab	27.49a	28.21a
Total nitrogen, %	1.65b	1.91a	1.93a	1.93a
Chloride, %	0.12	0.16	0.15	0.13
Potassium, %	2.70a	2.87a	2.24b	2.75a
Ratio of potassium to chloride, %	22.50a	17.97b	18.33b	17.23b
Ratio of reducing sugar to total sugar, %	84.28ab	80.78b	86.07a	84.57ab

Data presented as average value. Different lowercase letters indicate the significant difference of tobacco chemical composition among four agent addition treatments (*p* < 0.05).

### 3.2 Soil fungi abundance and alpha diversity

In total, 1,295,163 fungal ITS gene sequences passed quality control with sequences ranging from 82,255 to 94,227 ITS genes (Supplementary Table S1). After re-sampling, the number of OTUs ranged from 521 to 643 per sample for fungi. In addition, the coverage of all samples for fungi was above 99.9%, suggesting that the sequencing depth in this study was sufficient for diversity analysis (Supplementary Table S1). Venn analysis demonstrated that there were a total of 3,189 OTUs, and 1,250, 1,310, 1,168 and 1,201 OTUs in T1, T2, T3, and T4 soil, respectively (Supplementary Figure S1). Among that, there were 531, 647, 481, and 550 unique OTUs in. T1, T2, T3, and T4 soil, respectively (Supplementary Figure S1). These unique OTUs accounted for about 41.2–49.4% for all OTUs in four treatments (Supplementary Figure S1). In addition, there were 277 shared fungal OTUs, accounting for about 21.15–23.72% among four treatments (Supplementary Figure S1).

**Figure 1:**
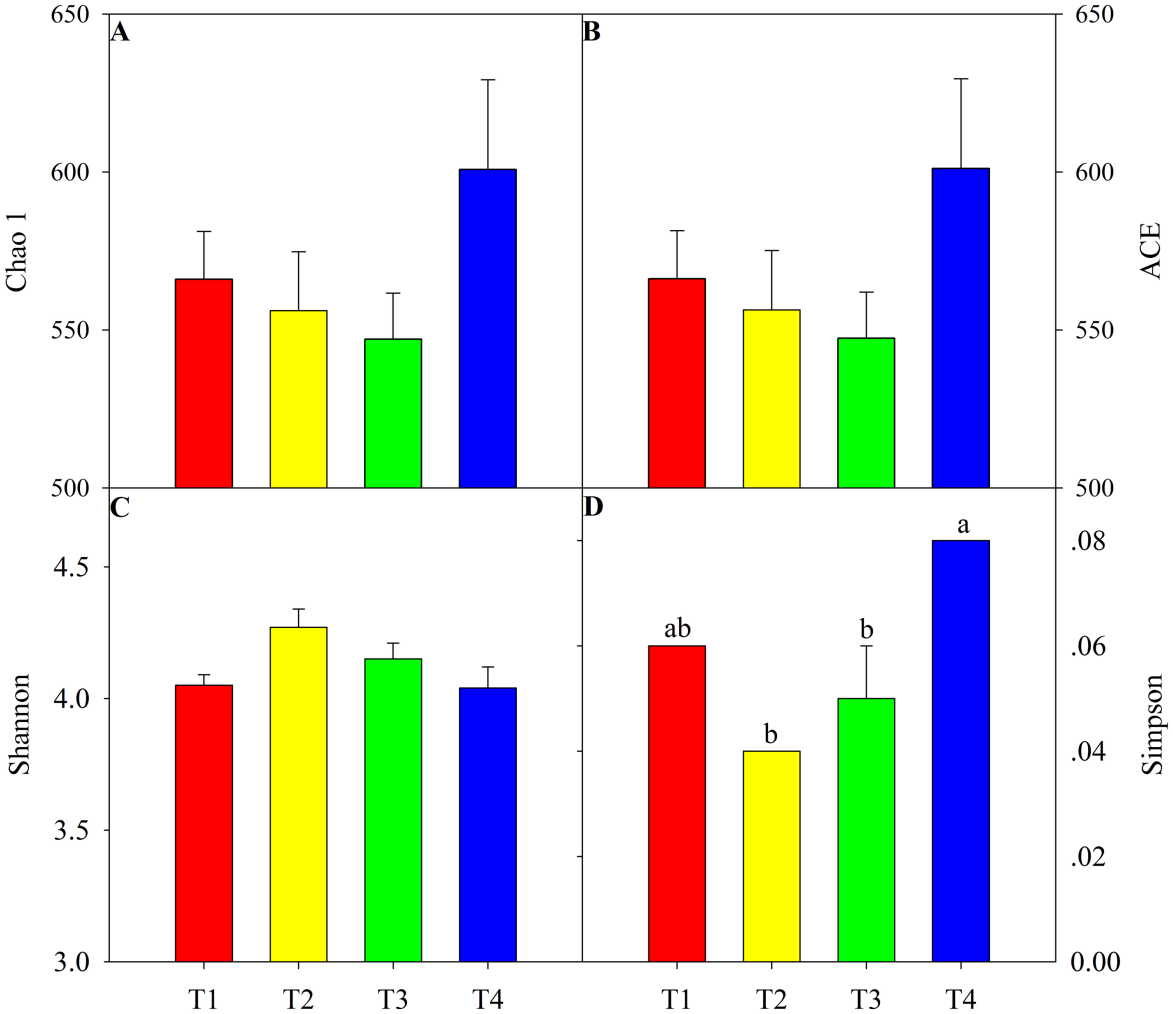
Soil fungi diversity under four agent addition treatments. Different lowercase letters present significant difference among four treatments at *p* < 0.05. T1: only chemical fertilizer, T2: microbial agent + chemical fertilizer, T3: lime + chemical fertilizer and T4: microbial agent + lime + chemical fertilizer.

**Figure 2:**
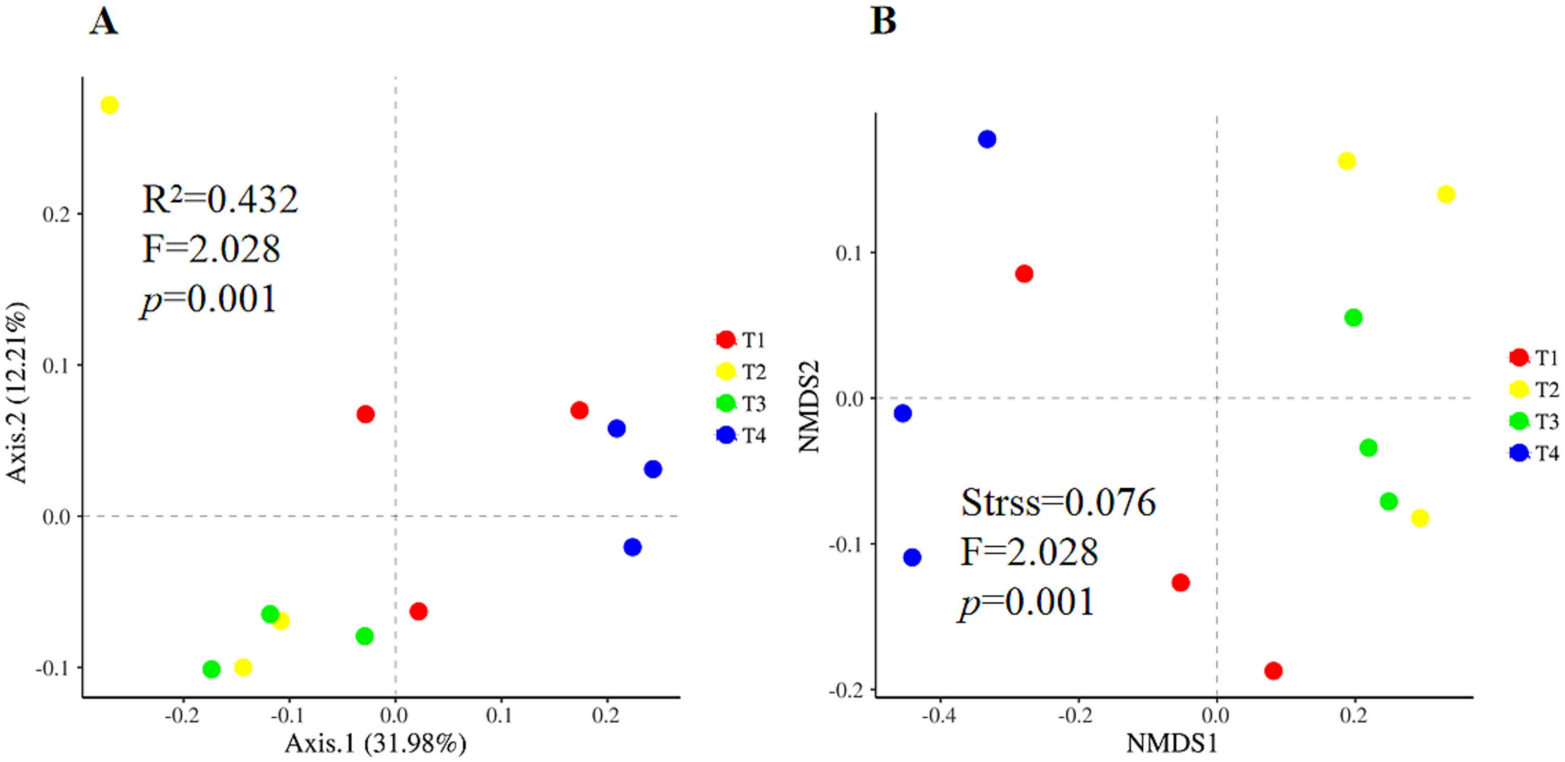
Beta diversity of soil fungal communities under four agent addition treatments. Principal coordinates analysis **(A)** and nonmetric multidimensional scaling **(B)** based on the Bray-Curtis distance dissimilarity.

According to the One-way ANOVA, certain alpha diversity indices, i.e., Simpson, were significantly shifted among four treatments. Compared with T1, T4 treatment significantly increased the Simpson index and the lowest value in T4 treatment (Figure 1D, *p* < 0.05). Chao 1 and ACE richness indices were similar to Simpson, with a higher value in T2 and lower in T4 but not achieving a significant level (Figures 1A,B, *p* > 0.05). Nevertheless, the Shannon indice of soil fungi was not significant different amonf four treatments (Figure 1C, *p* > 0.05). Principal coordinates analysis and nonmetric multidimensional scaling showed clear clustering of the fungal communities in different treatments (Figure 2). The first two principal components explained approximately 44.2% of the variance in the fungal composition based on the result of PCoA (Figure 2A). The aggregation degree of the sample point from the T3 and T4 soils was distant from those of the sample points from the T2 soil, and both soils were extremely far away from the sample points in the T1 soil (stress = 0.076, *F* = 2.03, *p* < 0.05, Figure 2B). Soil fungi in T3 and T4 treatment were clustered together and evidently separated from that in the other two treatments (Figure 2).

### 3.3 Soil fungal community structure and composition

**Figure 3:**
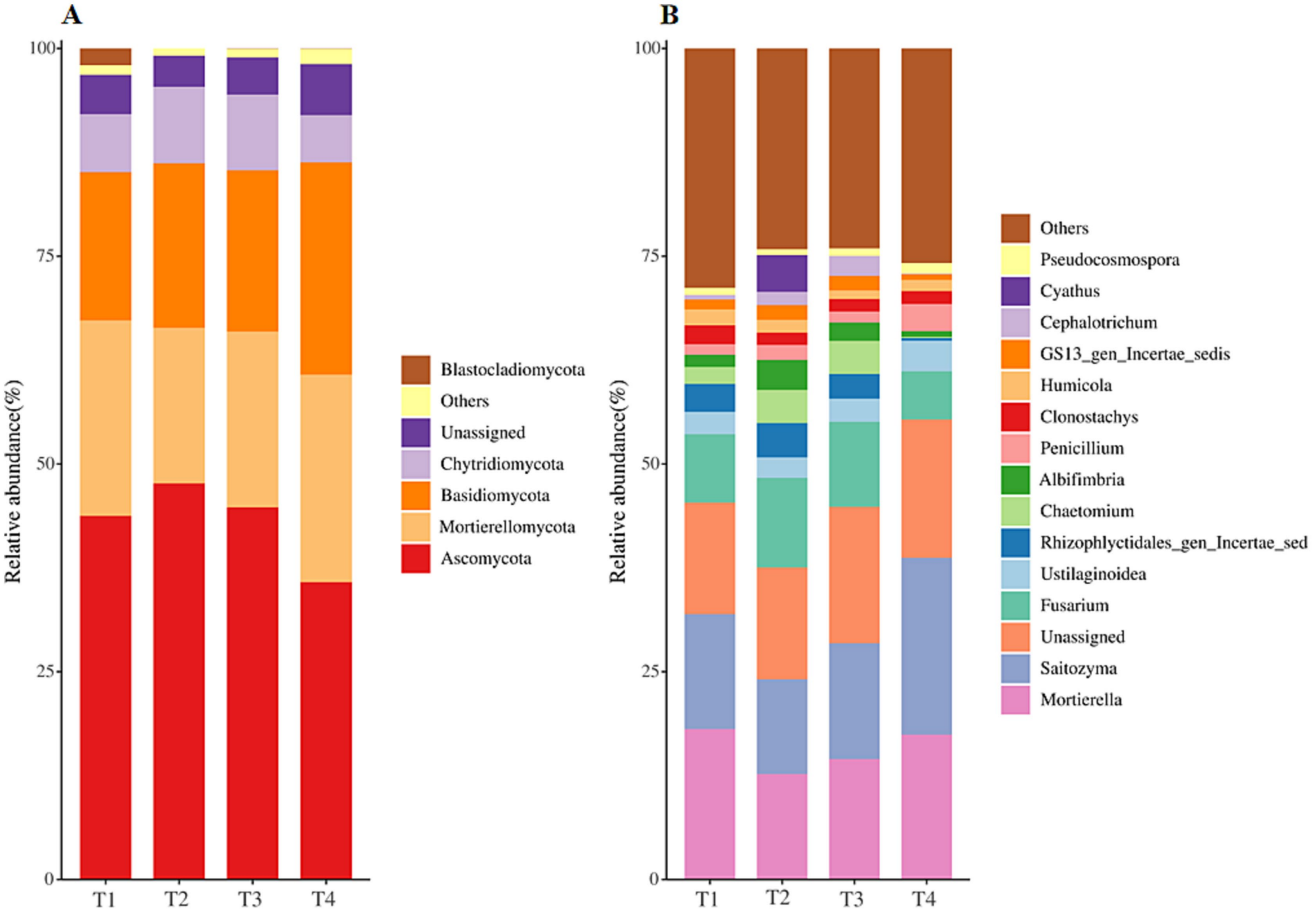
Relative abundance of dominant fungal phyla **(A)** and gene **(B)** among agent addition treatments.

The OTUs identified in all samples were divided into 15 phyla, 51 classes, 115 orders, 270 families, and 510 genes. Among four treatments, the overall fungal community was dominated by the phyla *Ascomycota* (average relative abundance >41.88%), followed by *Mortierellomycota* (average relative abundance 21.98%), *Basidiomycota* (average relative abundance 21.25%), and *Chytridiomycota* (average relative abundance 8.07%) (Figure 3A). The relative abundance of these dominant fungal phyla shifted differently to four treatments. For detail, the relative abundance of *Ascomycota* in the T4 treatment was significantly lower than that in the other three treatments (Figure 3A, *p* < 0.05), and there existed no significant difference in the relative abundance of *Ascomycota* in T1, T2 and T3 treatments (Figure 3A, *p* > 0.05). On the contrary, the relative abundance of *Basidiomycota* was significantly higher in T4 treatment and lower in T1 treatment (Figure 3A, *p* < 0.05). Further taxonomic classification at the gene level revealed that *Mortierella*, *Saitozyma* and *Unassigned* (average relative abundance above 15%) were the most abundant genes among all treatments, followed by *Fusarium* (average relative abundance 8.75%) and *Ustilaginoidea* (average relative abundance 3.0%) (Figure 3B). The relative abundance of *Unassigned*, *Saitozyma*, *Penicillium*, *Pseudocosmospora*, and *Mortierellales_gen_Incertae_sedis* was significantly increased while *Fusarium*, *Rhizophlyctidales_gen_Incertae_sedis*, *Chaetomium* and *Albifimbria* significantly decreased in T4 treatments when compared with those in T1 soil (Figure 3B and Supplementary Table S2, *p* < 0.05). The LEfSe was analyzed for discerning the abundant taxa among four fertilization types (Supplementary Figure S2). There are a total of 5, 15, 13 and 28 biomarkers in T1, T2, T3 and T4 soil, with all LDA values over 2.0. Furthermore, all biomarkers were completely different amongst soils, demonstrating that the fungal community structure of tobacco soil was induced by fertilization (Supplementary Figure S2).

The FUNGuild database can classify to predict the fungal community of soil under different rotation patterns. At least seven tropical modes were detected in this study and among, which saprotroph_symbiotroph modes were the most abundance (15.73% overall), followed by saprotroph, pathotroph_saprotroph and pathotroph modes (Supplementary Figure S3). The relative abundances of different fungal functional guilds also varied under the four different treatments. Specifically, compared with T1 soil, T4 treatment significantly increased the relative abundance of saprotroph fungi and decreased pathotroph_saprotroph fungi (Supplementary Figure S3, *p* < 0.05). Nevertheless, those two modes existed with an insignificant difference in T2 and T3 treatments related to those in T1 soils (Supplementary Figure S3, *p* > 0.05).

### 3.4 Relation between fungal community and soil properties

**Figure 4:**
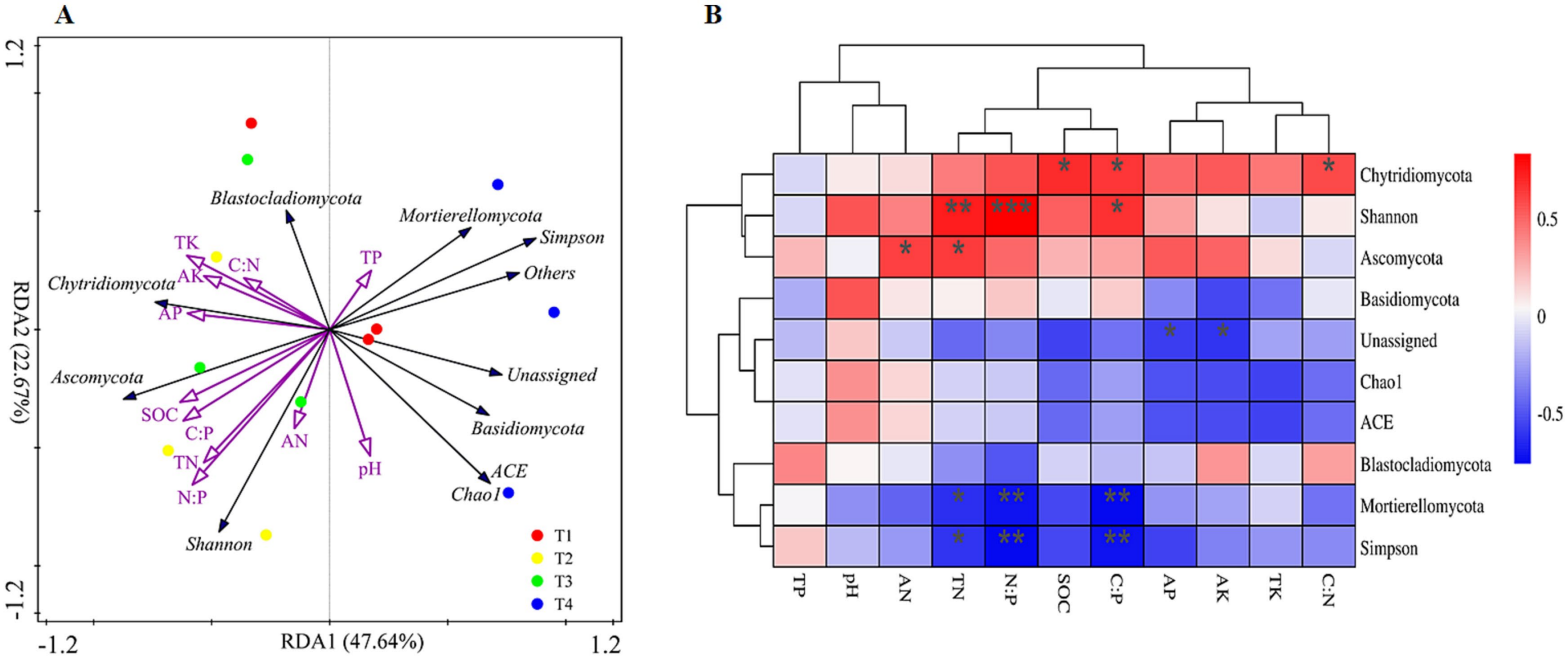
Redundancy analysis (RDA) diagram **(A)** and spearman’s rank correlations **(B)** illustrating the relationship between the soil fungal community composition (phyla level) and diversity from different sampling sites and soil properties. The explanatory variables are showed by different purple arrows, soil fungal community diversity and composition by black arrows.

The redundancy analysis model (RDA) was applied to analyze the relationships between soil properties and the fungal community compositions (Figure 4A). RDA1 and RDA2 explained 80.55% of the total variances, and all the soil properties clearly correlate with the fungal communities identified (Figure 4A). The RDA plot of the fungal community composition also clearly showed that the samples in T3 and T4 treatments significantly differed from the other two treatments (Figure 4A). The RDA model also revealed that SOC, TN, N:P, and AK play an important role in shaping fungi diversity and composition (Figure 4A). For diversity indices, Simpson was also negatively correlated with TN, C:P and N:P while Shannon was positively correlated with TN, C:P and N:P (Figure 4B, *p* < 0.05). For phylum level, the relative abundance of *Asocymota* was positively correlated with TN and AN while *Mortierellomycota* was negatively correlated with TN, C:P and N:P and unassigned phylum was negatively correlated with AP and AK (Figure 4B, *p* < 0.05). For gene level, SOC, TN, AP, C:P and N:P were clustered and significantly correlated with most fungal genes (Supplementary Figure S3, *p* < 0.05).

## 4 Discussion

### 4.1 Effect of agent amendments on improving soil fertility of tobacco soil

Our results demonstrated that the soil physicochemical properties were significantly impacted by the agent addition treatments. We believed that the variation of each soil property was likely to be affected by a single amendment or a combination of amendments that were added in our experiment. In general, the effectiveness of the agent on slowing soil acidification not only depends on the neutralization ability of hydrogen ion (H^+^) with lime addition but also on the mixed effect with microbial agent (Xiang et al., 2020; Kong et al., 2021; Liu et al., 2024). Compared with T1 treatment, soil pH value increased 0.46, 0.42, and 0.31 units in T2, T3 and T4 treatments, respectively, indicating the sole application of a microbial agent and/or together with lime has a positive effect on alleviating soil acidification.

We observed a significant increment in SOC content in all lime addition treatments compared to the T1 treatment. One possibility was probably due to more soil organic matter was stabilized by interaction with mineral surfaces via polyvalent cations (Di et al., 2015; Li et al., 2017). The polyvalent cation was calcium, mostly obtained via lime amendments. Furthermore, we discovered that the addition of microbial agents cause a large rise in SOC contents. Additionally, the microbial agent itself contains more SOC contents, and the addition of microbial agents can aid in the synthesis of soil organic matter by generating a range of enzymes (including urease, peroxidase, and catalase) through their life activities (Sun et al., 2017; Kong et al., 2021; Ahsan et al., 2024). These two scenarios could account for a notable increment in SOC content in all agent addition treatments. Owing to the coupled relationship between SOC and TN in soils, the content of soil TN also had significant increases in agent addition treatment, especially in T2 treatment. These results indicated that the combined application of microbial agents and/or microbial agents with mineral fertilization in this study sequestrated more nitrogen content than the sole mineral fertilization application. Above all, appropriate addition of microbial agents and lime in the short-term application can improve soil quality by alleviating soil acidification and increasing soil nutrient contents.

### 4.2 Effect of agent amendments on promoting tobacco growth and improving tobacco quality

In this study, the plant height, stem girth, pitch, length and width of maximum leaf, and dry matter weight of flue-cured tobacco leaf were notably higher in agent addition practices, mainly attributing to improved soil conditions induced by agent addition (Kong et al., 2021; Li et al., 2021; Li S. et al., 2022; Ahsan et al., 2024). The addition of microbial agent, either solely or in combination with lime, increases the level of the SOC, TN and pH, which enhances the ability of tobacco plants to absorb nutrients from the soil, hence facilitating the growth and reproduction of tobacco plants (Wu et al., 2014). Consequently, there currently is a considerable increase in tobacco yield when agent are added.

Chemical composition serves as the foundation for tobacco growth and development, as well as an influence on smoke qualities, which are critical to the quality and style of tobacco (Johnstone and Plimmer, 1959). The composition and coordination of the conventional chemical components are the primary factors relied on by the tobacco industry when selecting tobacco leaves (Chen et al., 2021). Total sugar, reducing sugar, total nitrogen, chlorine, and potassium contents in tobacco is frequently used to evaluate its intrinsic quality, scent, and taste (Song et al., 2007). Sugar, for example, plays an essential role in tobacco by participating in the synthesis of proteins, nucleic acids, lipids and other substances that supply energy for tobacco growth and development, as well as engage in the synthesis of aromatic chemicals (Banožić et al., 2020). Generally, high-level sugar tobaccos are usually desired by tobacco producers and consumers because sugar has a direct impact on the sensory quality of leaves, the quality of cut tobacco, and the aroma and flavor of smoking (Song et al., 2007). In this study, the addition of microbial agent with chemical fertilizer and lime alone or together enhanced the total sugar content of tobacco by 10.86–15.86% and the reducing sugar content by 10.26–16.19%, respectively, as compared to T1. Additionally, the highest level was found in T4, suggesting that the combination of microbial agent and lime with chemical fertilization was more effective in producing high-level sugar tobacco. Furthermore, all of the nitrogen and potassium content of tobacco leaves satisfies the standards of high-quality tobacco, which include total nitrogen levels above 1.5% and potassium content levels above 2%. Nonetheless, the overall nitrogen and potassium content of tobacco has significantly increased in T4. Considering other chemical indicators of tobacco leaves, in general, T4 treatment can generally promote the harmonization of chemical substances in tobacco leaves, including lowering the ratio of potassium to chlorine, increasing the content of total sugar, the reducing sugar, total nitrogen, and slightly increasing chlorine and potassium contents.

### 4.3 Changes in soil fungi community diversity and structure

An indicator of soil microbial community diversity is crucial for the productivity and stability of agricultural soil ecosystems (Sharma et al., 2011; Trivedi et al., 2016). Based on the findings of this study, it is noteworthy to highlight that soil fungus community richness in all agent addition treatments was comparable to that in the T1 treatment. The comparatively higher Shannon and lower Simpson values in T4 treatment provided additional indication that fungi in T4 soil were more variety. This heightened vulnerability can be attributed to their exceptional capacity to acquire organic nitrogen and phosphorus, as well as their vital functions in nutrient cycling within the soil ecosystem (Richardson et al., 2009). Similarly, this study indicated that the structure and diversity of soil fungi are highly impacted by soil TN, N:P, and C:P. The finding can be related to differences in soil properties brought about by various agricultural practices, which affect the availability of microbial nutritional elements. Consequently, these elements operate as cofactors in the synthesis of numerous organic substances, such as acids, proteins, and carbohydrates, which modified the composition of the soil microbiome (Liu et al., 2023). Beta diversity further indicated that fungi community composition was divided into three separate clusters based on agent addition types. In our study, fungi diversity was found to be more sensitive to the joint application of agent addition and mineral fertilization than to the sole mineral fertilization practice. This suggested that the microbial agent may be a determinant driving factor affecting fungi community composition, which has been verified by other researches (Kong et al., 2021; Xin et al., 2022; Wu et al., 2023; Ahsan et al., 2024). They indicated that the application of microbial agents has the potential to improve the abundance, community composition, and functional diversity of microorganisms dwelling in soil.

The effect of agent addition and fertilization practice on the soil fungal community composition was distinctly observed at the phylum and genus level. Generally, the phylum *Ascomycota* was more likely to be formed in diseased soil and to be associated with a wide range of crop monoculture systems (Yuan et al., 2020; Zhou et al., 2023). In our study, *Ascomycota* had a higher abundance in tobacco planting soil, and its relative abundance in T4 soil (average 35.77%) lower than that in T1 soil (average 43.75%), indicating that combination of microbial agent with lime application and chemical fertilizer is an improved strategy to defend crops against specific disease like *Fusarium* wilt disease (Shen et al., 2015). In addition, the *Ascomycota* class *Sordariomycetes* is the most dominant (top 1 class, average relative abundance = 31.92%), which is consistent with numerous studies showing *Sordariomycetes* to be the most prevalent fungal class in a variety of agricultural systems (Xue and Liu, 2021). Members of this class, including *Fusarium*, *Ustilaginoidea*, *Chaetomium*, *Albifimbria*, *Clonostachys* and *Humicola* gene, have a broad distribution as plant endophytes and pathogens in almost all ecosystems (Sun et al., 2020). These substance are typically supposed to be the main plant pathogens (Li et al., 2014; Shen et al., 2015).

Agent addition has been demonstrated to have huge impacts on the general distribution and prevalence of fungal abundance, that involve a broad range of functional species (Ahsan et al., 2024). The reduction of pathogenic and accumulation of potential beneficial communities might help to reduce plant disease caused by continuous cropping barrier (Wen et al., 2024). In general, saprotrophic fungi are the primary decomposers in the soil, its enrichment in soil may restrict soil-borne fungal pathogens (Wen et al., 2024). Compared with T1, T2, and T3 treatment reduced the abundance of saprotrophic fungi, while the application of T4 significantly increased saprotrophic fungi, which would reduce the incidence of diseases and thus improve plant growth. *Fusarium* is recognized as a pathogenic fungus that causes devastating crop diseases as *Fusarium* root rot in tobacco crops (Liu et al., 2019; Gai et al., 2023; Liang et al., 2024). In this study, *Fusarium* was the most prevalent pathogenic fungi among all treatments. Among three agent application treatments, only T4 reduced *Fusarium* abundance as compared to T1. Furthermore, other potential plant pathogenic fungi such as *Alternaria*, which may infect various crops and cause foolish seeding disease, soybean *Altenaria* leaf spot, and citrus *Alternaria* brown spot (Li et al., 2014; Wang et al., 2021) was also reduced in T4 treatment. Our findings revealed that certain potential beneficial species, e.g., *Penicillium* and *Trichoderma*, were enriched after agent addition. These species have the capacity to promote plant-growth and enhance plants resistance to biotic and abiotic stresses (Hermosa et al., 2012; Sennoi et al., 2013; Altaf et al., 2018; Chaudhary et al., 2018; Srinivasan et al., 2020). For example, *Penicillium* species interact positively with roots of crop plants and enhance the plant growth by supplying nutrients and plant growth hormones like indole-3-acetic acid and gibberellic acid, which are important in suppressing *Fusarium* wilt and take-all diseases (Srinivasan et al., 2020). López-Bucio et al. (2015) also demonstrated that *Trichoderma* help plants better resist environmental stresses such as salinity and drought via reinforcing plant growth and reprograming gene expression in roots and shoots. In our study, these genes were more prevalent in T4 treatment than in T1. Thus, our findings suggest that combining microbial agents and lime with chemical fertilizer may improve the quantify of beneficial fungi while lessening the prevalence of pathogenic fungi. As consequence, it may be extremely crucial in alleviating the barrier associated with chemical fertilization solely in tobacco cultivation.

## 5 Conclusion

Our study indicates that different agent addition strategies had divergent effects on soil physicochemical properties and microbial community structure of tobacco cultivation. In comparison to using only chemical fertilization in tobacco cultivation, the application of microbial agents and/or lime in conjunction with chemical fertilizer greatly increased soil nutrients and tobacco yields, as well as the chemical composition of tobacco leaves. Agent amendment applications also distinctly shifted the soil fungi community diversity. *Ascomycota* was the most prevalent phylum among all treatments. T4 (combination application of lime and microbial agents with chemical fertilization) treatment clearly accumulated some beneficial fungi and significantly reduced or did not detect certain potentially pathogenic fungi when compared to solely chemical fertilization. Furthermore, the FUNGuild function predicts an explicit enrichment of saprotrophic fungi in T4 therapy, preventing the prevalence of potential pathotypes in symbionts. The fungal community structure was extremely significant and relevant in relation to soil nutrients. On the whole, we proposed that combination application of microbial agents and lime with chemical fertilizer could be an effective strategy for promoting efficient tobacco cultivation, given that it eliminates soil acidification, improves soil nutrients and modifies the soil microbial community structure. Furthermore, more study on long-term agent addition is required to acquire a comprehensive understanding of the dynamic modifications occurring within microbial communities and functional genes in regions where tobacco is continuously cultivated.

## Data Availability

The complete datasets generated in our study have been submitted in NBCI Sequence Read Archive database (BioProject and accession: PRJNA1238546).
