# Supplementary material for: Combination application of microbial agent and lime application improves soil nutrients and soil fungi community, producing good quality tobacco
Source: Front Microbiol. 2025 Jul 22;16:1615412. doi: 10.3389/fmicb.2025.1615412 (PMC12321893; doi:10.3389/fmicb.2025.1615412)
Supplement: Supplementary file 1 [file Data_Sheet_1.doc]

**Table S1** Soil microbial sequence number

| Sample | Input | Filtered | Input passed filter (%) | Denoised | Merged | Input merged (%) | Non-chimeric | Input non-chimeric(%) | Coverage |
| --- | --- | --- | --- | --- | --- | --- | --- | --- | --- |
| T1-1 | 83627 | 71518 | 85.52 | 70778 | 65633 | 78.48 | 65606 | 78.45 | 0.9999843 |
| T1-2 | 82661 | 68798 | 83.23 | 67936 | 64696 | 78.27 | 64559 | 78.1 | 1 |
| T1-3 | 86597 | 73073 | 84.38 | 72403 | 68334 | 78.91 | 68303 | 78.87 | 0.9999686 |
| T2-1 | 86239 | 70682 | 81.96 | 70083 | 63662 | 73.82 | 63615 | 73.77 | 1 |
| T2-2 | 84082 | 71835 | 85.43 | 71325 | 67296 | 80.04 | 67270 | 80.01 | 0.9999686 |
| T2-3 | 86132 | 72460 | 84.13 | 71766 | 67302 | 78.14 | 67176 | 77.99 | 0.9999529 |
| T3-1 | 88208 | 73525 | 83.35 | 72859 | 67510 | 76.54 | 67464 | 76.48 | 0.9999686 |
| T3-2 | 85368 | 71186 | 83.39 | 70602 | 66449 | 77.84 | 66446 | 77.83 | 0.9999686 |
| T3-3 | 82255 | 69786 | 84.84 | 69217 | 65390 | 79.5 | 65330 | 79.42 | 0.9999686 |
| T4-1 | 85573 | 72911 | 85.2 | 72291 | 66801 | 78.06 | 66766 | 78.02 | 0.9999843 |
| T4-2 | 91146 | 77208 | 84.71 | 76529 | 69186 | 75.91 | 69069 | 75.78 | 0.9999371 |
| T4-3 | 89167 | 73210 | 82.1 | 72483 | 66709 | 74.81 | 66528 | 74.61 | 0.9999686 |

T1: only chemical fertilizer, T2: microbial agent + chemical fertilizer, T3: lime + chemical fertilizer and T4: microbial agent + lime + chemical fertilizer. The same below.

**Table S2** Relative abundance (%) of fungal genera in different treatments with relative abundance greater than 0.5%

| phylum | Genus | T1 | T2 | T3 | T4 |
| --- | --- | --- | --- | --- | --- |
| *Ascomycota* | *Fusarium* | 8.23(0.15)ab | 10.77(0.07)a | 10.22(0.08)a | 5.8(0.05)b |
|  | *Ustilaginoidea* | 2.69(0.1) | 2.47(0.03) | 2.79(0.03) | 3.69(0.06) |
|  | *Chaetomium* | 2.04(0.08)ab | 3.98(0.10)a | 3.98(0.07)a | 0.15(0.01)b |
|  | *Albifimbria* | 1.46(0.05)b | 3.61(0.07)b | 2.20(0.00)b | 0.68(0.01)b |
|  | *Penicillium* | 1.28(0.02)b | 1.81(0.03)ab | 1.34(0.02)b | 3.27(0.10)a |
|  | *Clonostachys* | 2.27(0.05) | 1.48(0.04) | 1.50(0.02) | 1.55(0.05) |
|  | *Humicola* | 1.93(0.03) | 1.54(0.03) | 1.03(0.03) | 1.34(0.02) |
|  | *Cephalotrichum* | 0.5690.00)b | 1.58(0.07)ab | 2.40(0.05)a | 0.08(0.00)b |
|  | *Pseudocosmospora* | 0.84(0.02) | 0.69(0.03) | 0.91(0.03) | 1.27(0.01) |
|  | *Aspergillus* | 1.27(0.04) | 1.13(0.06) | 0.35(0.01) | 0.73(0.03) |
|  | *Fusicolla* | 0.99(0.03) | 0.86(0.01) | 0.75(0.01) | 0.86(0.01) |
|  | *Alternaria* | 0.53(0.01)b | 1.58(0.06)a | 0.59(0.02)ab | 0.27(0.00)b |
|  | *Cumuliphoma* | 0.82(0.02) | 0.55(0.01) | 1.26(0.04) | 0.34(0.00) |
|  | *Lecythophora* | 0.66(0.01) | 0.64(0.03) | 0.84(0.03) | 0.57(0.02) |
|  | *Talaromyces* | 1.32(0.08) | 0.18(0.01) | 0.45(0.02) | 0.74(0.03) |
|  | *Pseudothielavia* | 1.14(0.08) | 0.67(0.01) | 0.62(0.01) | 0.16(0.01) |
|  | *Pseudeurotium* | 0.37(0.01) | 0.86(0.04) | 0.57(0.02) | 0.49(0.01) |
|  | *Neocosmospora* | 0.85(0.02) | 0.48(0.01) | 0.39(0.01) | 0.55(0.02) |
|  | *Trichoderma* | 0.43(0.00)b | 0.22(0.00)b | 0.3190.00)b | 0.98(0.01)a |
|  | *Marquandomyces* | 0.21(0.01)b | 0.71(0.01)a | 0.63(0.01)a | 0.03(0.00)b |
|  | *Minutisphaera* | 0.16(0.01)b | 0.20(0.02)b | 0.08(0.00)b | 1.11(0.00)a |
|  | *Sordariales_gen_Incertae_sedis* | 1.48(0.15) | 0 | 0 | 0 |
|  | *Paraphaeosphaeria* | 0.61(0.02)a | 0.10(0.00)b | 0.09(0.00)b | 0.49(0.01)ab |
| *Mortierellomycota* | *Mortierella* | 18.09(0.20) | 12.69(0.12) | 14.48(0.21) | 17.43(0.22) |
|  | *Linnemannia* | 0.76(0.03) | 0.91(0.03) | 0.81(0.03) | 1.1(0.03) |
|  | *Mortierellales_gen_Incertae_sedis* | 0.65(0.01)b | 0.38(0.02)b | 0.42(0.01)b | 1.63(0.03)a |
|  | *Mortierellaceae_gen_Incertae_sedis* | 0.06(0.00)b | 0.02(0.00)b | 0.05(0.00)b | 0.31(0.01)a |
| *Basidiomycota* | *Saitozyma* | 13.83(0.04)b | 11.43(0.20)b | 13.93(0.14)b | 21.3(0.13)a |
|  | *Cyathus* | 0 | 4.43(0.44) | 0 | 0 |

Data was presented as means ± standard error. Different lowcase letters means significant difference among different treatments (*p*<0.05).


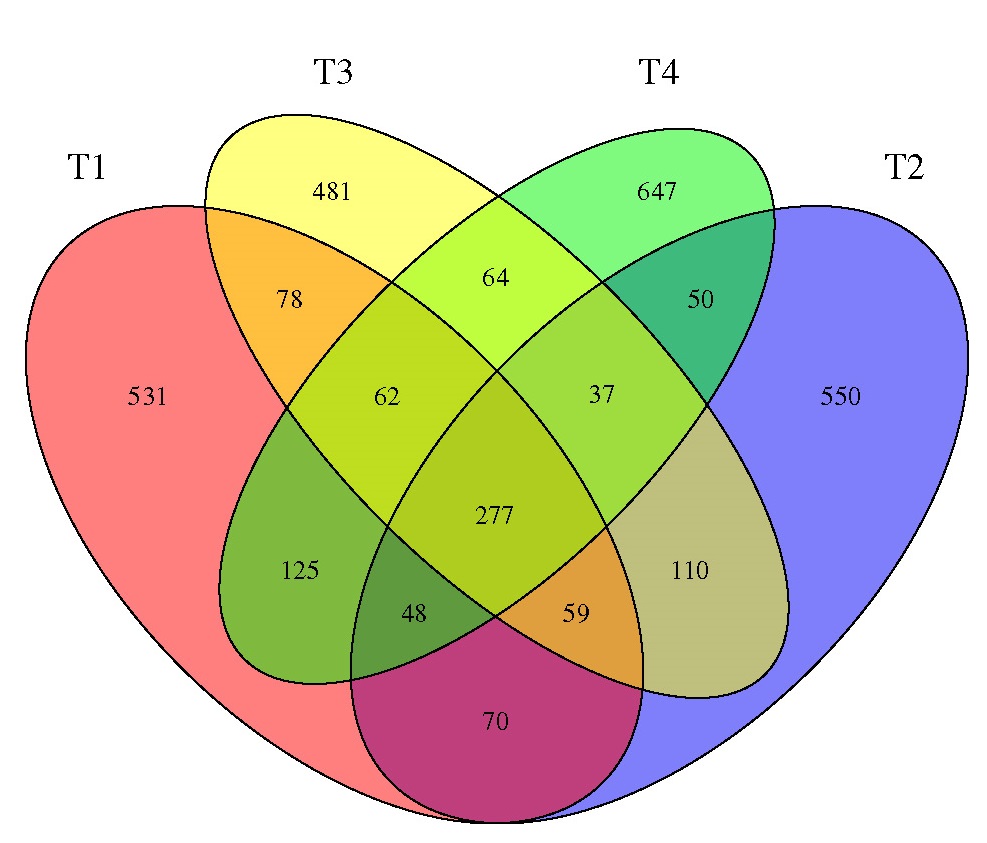


**Figure S1** The Venn of soil fungi community among four agent addition treatments. T1: only chemical fertilizer, T2: microbial agent + chemical fertilizer, T3: lime + chemical fertilizer and T4: microbial agent + lime + chemical fertilizer. The same below.





**Figure S2** Biomarkers with statistical differences among four agent addition treatments were determined by LEfSe.





**Figure S3** The distribution of FUNGuild among four treatments.


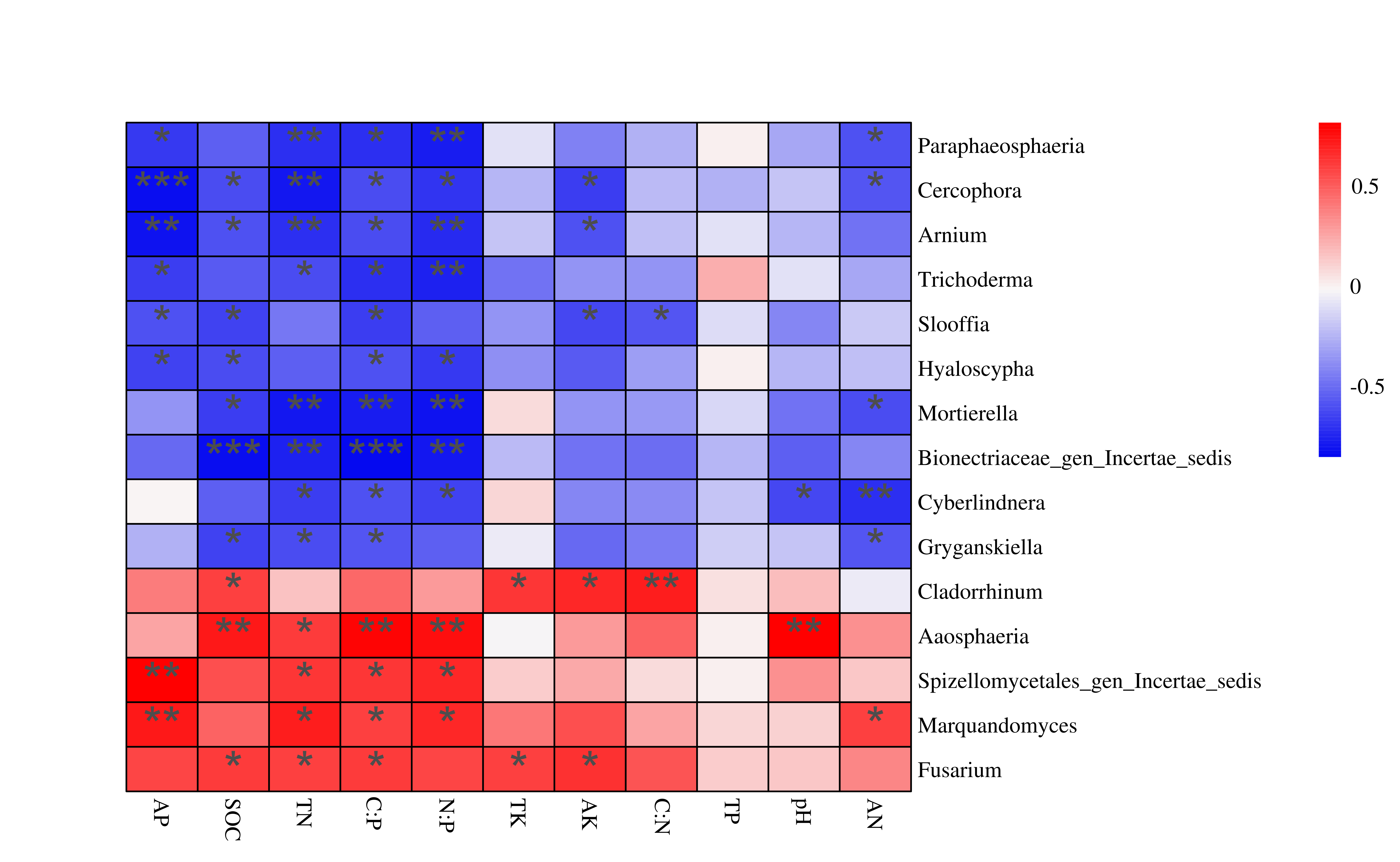


**Figure S4** Spearman’s rank correlations showing the relationship between soil fungal community composition (gene level) and soil properties
